# Unraveling the Connection: Visceral Adipose Tissue and Vitamin D Levels in Obesity

**DOI:** 10.3390/nu15194259

**Published:** 2023-10-05

**Authors:** Mattia Cominacini, Alessia Fumaneri, Linda Ballerini, Michele Braggio, Maria Teresa Valenti, Luca Dalle Carbonare

**Affiliations:** 1 Section of Biomedicine, Department of Engineering for Innovation Medicine, University of Verona, 37134 Verona, Italy; mattia.cominacini@univr.it (M.C.); alessia.fumaneri@gmail.com (A.F.); linda.ballerini@gmail.com (L.B.); michele.braggio@gmail.com (M.B.); luca.dallecarbonare@univr.it (L.D.C.); 2 Department of Neurosciences, Biomedicine and Movement Sciences, University of Verona, 37134 Verona, Italy

**Keywords:** obesity, storage, ultrasound, visceral adipose tissue, vitamin D, waist circumference

## Abstract

Vitamin D deficiency and insufficiency are widespread on a global scale, with multiple factors playing a role in their development, such as limited exposure to sunlight, inadequate dietary consumption, as well as obesity and abdominal fat accumulation. Abdominal obesity, assessed with waist circumference (WC), is associated with metabolic syndrome and has been linked to low vitamin D levels. This study aimed to investigate the relationship between visceral adipose tissue (VAT) and vitamin D levels, particularly examining the potential threshold for vitamin D storage and sequestration using adipose tissue. The study was conducted between 2020 and 2022 with 58 patients from an internal medicine outpatient department. Patients with certain medical conditions and those taking medications affecting bone metabolism were excluded. Blood samples were collected at baseline and after 6 months of monthly cholecalciferol supplementation. Ultrasonography was used to evaluate adipose tissue measurements, including subcutaneous adipose tissue thickness, VAT, preperitoneal adipose tissue (PPAT), and prerenal adipose tissue (PRAT). Anthropometric measures such as the waist-to-hip ratio and waist-to-height ratio were also assessed. The results showed that all subjects had significant hypovitaminosis D at baseline. After 6 months of supplementation, the mean increase in vitamin D levels was 9.6 ng/mL, with 55.2% of subjects becoming deficient. The study revealed a significant correlation between follow-up vitamin D levels and waist circumference, hip circumference, and VAT. VAT exhibited a strong correlation not only with vitamin D levels but also with waist circumference. When analyzing gender differences, males showed a higher weight and waist-to-hip ratio, while females had higher body adiposity indexes and subcutaneous adipose tissue measurements. In conclusion, this study highlights the relationship between VAT and vitamin D levels, emphasizing the potential role of adipose tissue in vitamin D availability. Waist circumference was identified as a surrogate measure for VAT evaluation. Furthermore, the study showed variations in vitamin D response to supplementation between genders, with a higher percentage of males reaching normal vitamin D levels. Predictive factors for vitamin D levels differed between genders, with waist circumference being a significant predictor in males and body adiposity index in females.

## 1. Introduction

Vitamin D deficiency and insufficiency have emerged as a significant global health concern, affecting populations across the world [1]. These conditions arise from various factors, with the major circulating form of serum vitamin D, known as 25-hydroxy vitamin D (25 [OH] D), playing a pivotal role in metabolic processes. Low outdoor physical activity and subsequent low sun exposure, poor dietary intake of vitamin D, obesity, and especially abdominal obesity are involved in the etiology of hypovitaminosis D [2,3]. In particular, one of the primary factors contributing to vitamin D deficiency is the modern lifestyle characterized with low outdoor physical activity and subsequent diminished sun exposure. In an era marked with sedentary occupations and indoor-centric leisure activities, individuals are spending less time outdoors, leading to reduced opportunities for their skin to synthesize vitamin D in response to sunlight. Dietary habits also play a crucial role in the etiology of vitamin D deficiency. Many people fail to obtain an adequate intake of vitamin D-rich foods, such as fatty fish, fortified dairy products, and fortified cereals. This nutritional deficit can be further exacerbated with specific dietary preferences and restrictions, which may limit the variety of vitamin D sources in one’s diet. Addressing the worldwide prevalence of vitamin D deficiency and insufficiency necessitates a multifaceted approach. Public health initiatives should focus on promoting outdoor physical activity and sun exposure, particularly in regions with limited sunlight during certain seasons. Nutrition education programs can help individuals make informed dietary choices to increase their vitamin D intake. Additionally, healthcare professionals should be vigilant in assessing the vitamin D status of patients, especially those with obesity-related conditions, and recommend appropriate supplementation when necessary.

Obesity, especially abdominal obesity, is another significant contributor to hypovitaminosis D. Fat-soluble vitamin D is sequestered in adipose tissue, which can reduce its bioavailability in the bloodstream. Furthermore, obese individuals often experience metabolic disturbances that affect the conversion of vitamin D into its active form, exacerbating the deficiency. Abdominal obesity, typically assessed using waist circumference (WC) measurements, constitutes a key element of metabolic syndrome. Previous investigations have uncovered a noteworthy association between obesity, particularly abdominal obesity, and lower serum vitamin D levels. Several authors have reported a significant inverse relationship between abdominal obesity and serum vitamin D levels [4,5,6,7,8]. Furthermore, research has indicated that visceral adiposity elevates the risk of systemic inflammation, insulin resistance, diabetes mellitus, and cardiovascular diseases [9,10,11,12,13]. The observed negative correlation between vitamin D levels and WC underscores the importance of maintaining both adipose tissue and vitamin D levels within the normal range to prevent metabolic disorders, even in children [14,15,16]. Obese children experienced a 45% reduction after equal doses of vitamin D administration. This deficiency can be addressed by increasing vitamin D doses two-fold or even three-fold, with doses up to 10,000 IU daily considered safe for most patients. Regular monitoring of 25(OH)D values is recommended for overweight or obese individuals to ensure optimal vitamin D sufficiency [17,18]. However, it has been observed that different doses of cholecalciferol are needed to normalize vitamin D levels in various situations and types of individuals [19]. Vitamin D exerts an effect on adipogenesis, apoptosis, oxidative stress, inflammation, the secretion of adipocytokines, lipid metabolism, and thermogenesis, and contributes to the maintenance of adipose tissue structure, function, and fat content [20,21]. The relationship between vitamin D and adipose tissue is intricate, and the role of adipose tissue in regulating circulating vitamin D levels is not clear, particularly whether it acts as a reservoir or sequestration site for the vitamin. Certain researchers have noted that the expanded fat tissue in obese individuals can function as a storage site for vitamin D. This heightened demand for vitamin D to fill this reservoir may potentially lead to insufficient levels of serum vitamin D in obese individuals [22,23]. From another point of view, some authors argue that adipose tissue may sequester vitamin D, reducing its bioavailability [24]. Other authors suggest that reduced levels of 25-hydroxyvitamin D are linked to an increase in 1,25-dihydroxyvitamin D levels in obese individuals [25,26], but this interpretation is not shared by other authors who have observed opposite results [27,28,29].

To elucidate the actual relationship between vitamin D levels and adipose tissue, especially visceral adipose tissue, it may be beneficial to employ a more precise approach for adipose tissue assessment as opposed to the mere calculation of BMI or waist circumference measurement. Ultrasonography is becoming the gold standard for the evaluation of many anatomic areas and even for the study of visceral adipose tissue, it is considered an accurate approach [30]. In particular, it has been observed that there is an optimal correlation between ultrasound parameters of VAT and computed tomography (CT) [31]. Using this approach, the cut-off value of visceral fat area for central obesity and metabolic syndrome was found to be 100 cm [31,32,33]. The possibility to evaluate visceral adipose tissue using a non-invasive approach can clarify the effective role of adipose tissue in managing vitamin D availability.

Thus, the central focus of our study was to elucidate whether there exists a specific threshold or critical point at which the storage and metabolism of vitamin D within the human body are significantly influenced by the presence of visceral adipose tissue. This threshold, if identified, would hold valuable implications for understanding the dynamics of vitamin D homeostasis and potentially offer insights into novel approaches for managing vitamin D-related health concerns. In fact, our research was motivated by the recognition that vitamin D, a vital fat-soluble micronutrient, plays an indispensable role in various physiological processes, and its proper regulation within the body is paramount for maintaining overall health. In the pursuit of this scientific endeavor, our study embarked upon an in-depth exploration of the intricate interplay between visceral adipose tissue, which refers to the adipose deposits located deep within the abdominal cavity, and the levels of vitamin D circulating in the bloodstream.

The findings of this research hold the promise of advancing our understanding of vitamin D metabolism and its relevance to human health, paving the way for further investigations and potentially innovative approaches to optimizing vitamin D status.

## 2. Methods

### Patients

This longitudinal observational study was conducted during a period between 2020 and 2022; the group was composed of 58 patients consecutively enrolled in the internal medicine outpatient department of Azienda Ospedaliera Universitaria Integrata of Verona. The inclusion criteria were Caucasian ethnicity, presence of an overweight status or obesity, according to BMI values (BMI ≥ 25), and vitamin D levels less than 30 mg/mL.

Patients taking drugs that could alter bone metabolism such as corticosteroids, antiepileptics, and thyroid hormones were excluded from the study. In the same way, patients affected by metabolic diseases such as primary hyperparathyroidism, chronic renal failure, and liver failure were excluded from the study.

All participants were informed about the details of the research and signed the informed consent before the enrolment. The study was registered in the Clinical Trial Registry (Clinicaltrial.gov ID: NCT05957692). The protocol for sample collection was approved by the ethical committee of Azienda Ospedaliera Universitaria Integrata of Verona, Italy (number 1538; 3 December 2012; local ethical committee of Azienda Ospedaliera Integrata di Verona). The study complied with the revised ethical guidelines of the Declaration of Helsinki.

All subjects underwent blood sampling to evaluate calcium and vitamin D levels at baseline and after 6 months of supplementation with 50,000 IU of cholecalciferol per month. Using ultrasonography, we evaluated at baseline and after 6 months of vitamin D supplementation subcutaneous adipose tissue thickness minimum and maximum (SAT), visceral adipose tissue (VAT), preperitoneal adipose tissue (PPAT), prerenal adipose tissue (PRAT) waist-to-hip ratio, and waist-to-height ratio. In addition, we calculated the body adiposity index (BAI) as the result of the following mathematical equation: hip circumference (cm)/height (m) 1.5–18 [34].

## 3. Statistical Analysis

All statistical analyses were performed using Windows SPSS software, version 22.0 (SPSS Inc., Chicago, IL, USA) and Jamovi software, version 2.3.21. The results obtained were expressed as the mean ± Standard Deviation.

Sample size was calculated using the statistical software PASS 14.0.8 (Kaysville, UT, USA) with the “test for paired means”, considering a potency of 90% and an alpha error of 5%.

To compare group differences, we employed the paired T-Student test and multifactorial analysis of variance (ANOVA). When the data did not follow a normal distribution, non-parametric tests, such as the Mann–Whitney U test, were utilized.

We assessed potential linear relationships between variables using the Pearson coefficient and explored potential predictive factors through multivariate linear regression tests for continuous variables and logistic regression for categorical variables. Additionally, we conducted an ROC analysis to determine the optimal threshold for key determinants of vitamin D levels. Statistical significance was determined at a *p*-value threshold of <0.05.

## 4. Results

### Population of the Study

**Figure 1:**
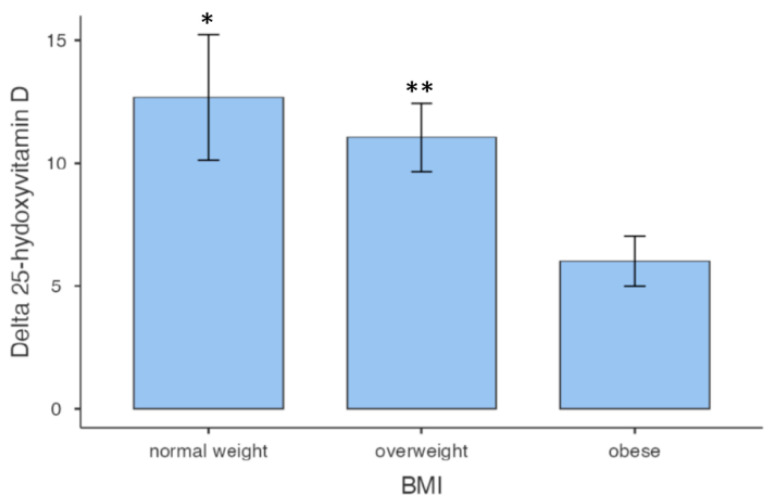
Vitamin D variations among BMI classes. * *p* = 0.023 vs. obese; ** *p* = 0.046 vs. obese.

A total number of 58 subjects were included in the study, 43 male (71%) and 15 female (29%). The general characteristics, the biometric measures, and 25 [OH] D pre-treatment concentration of all subjects are described in Table 1. All subjects showed significant hypovitaminosis D, compatible with insufficiency. Only 13 (22.4%) subjects had normal weight, 25 (43.1%) were overweight, and 20 (34.5%) were obese. Classifying patients based on BMI, we observed that in obese individuals, the increase in vitamin D at the end of the study was approximately half of that in normal-weight and overweight subjects (Figure 1).

Regarding waist circumference, no women and only two men had normal levels; all other subjects exhibited values indicative of abdominal obesity. Concerning cardiovascular diseases, 41 (70.7%) had hypertension, 14 (24.1%) had diabetes, 26 (44.8%) had hypercholesterolemia, 47 (81%) had fatty liver disease, 35 (60.3%) had atrial fibrillation, and 13 (22.4%) had venous thromboembolic events (DVT or PE). The mean daily alcohol intake was higher or equal to 2 units in 35 (60.4%) subjects. None of the subjects were smokers.

After 6 months of 50,000 IU of cholecalciferol supplementation per month, the mean 25 [OH] D concentration was 27.3 ± 6.2 ng/mL and the mean increase was 9.6 ± 7.3 ng/mL. 25 [OH] D remained insufficient in 8 subjects (13.8%) while 32 (55.2%) became deficient and 18 (31%) reached normal vitamin D levels.

**Figure 2:**
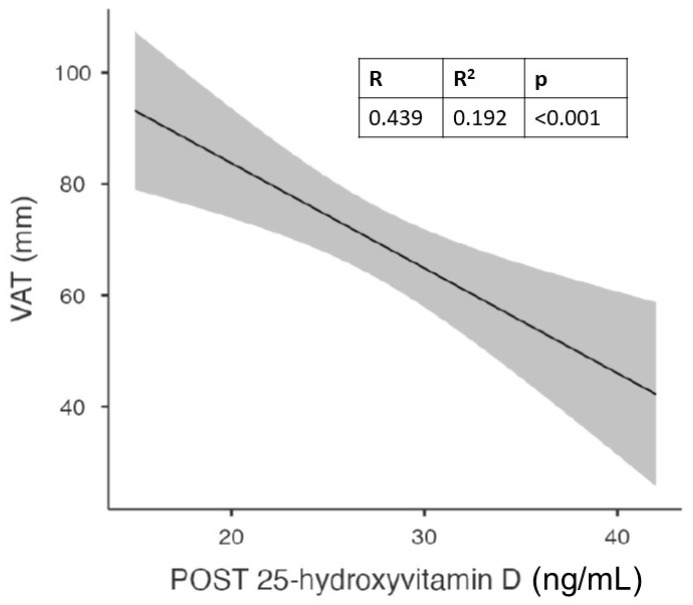
Correlation between visceral adipose tissue (VAT) and vitamin D levels after 6 months of cholecalciferol in the whole population.

First of all, we wanted to analyze the population as a whole, considering only the variables that did not show significant differences between the two genders (Figure 2).

With this analysis, we found a significant correlation between follow-up vitamin D values and waist circumference, hip circumference, and visceral adipose tissue. The latter showed a strong and statistically significant correlation not only with vitamin D levels but also with waist circumference.

BMI (body mass index) and BAI (body adiposity index) were excluded from the analysis of the overall study population as they are derived variables significantly different in both genders from both height and weight.

Figure 1 shows the significant correlation between visceral adipose tissue (VAT) and vitamin D levels after 6 months of cholecalciferol in the whole population.

When we grouped subjects according to gender, we found a significantly higher weight and waist-to-hip ratio in males, while mean SAT was higher in females (Table 2).

In addition, in the whole population, we observed a significant correlation between VAT and waist circumference, as previously reported [35,36,37]. This suggests how the measurement of waist circumference can be considered a good surrogate for the assessment of visceral adipose tissue.

Given the different distribution of body fat, we wanted to analyze all anthropometric variables separately in males and females.

**Figure 3:**
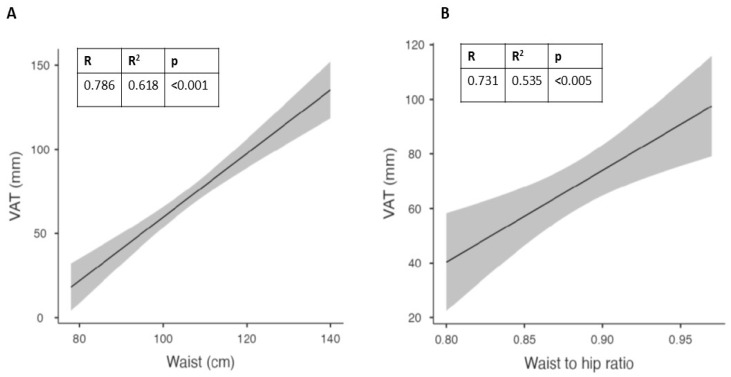
Best correlations at baseline between visceral adipose tissue (VAT) and waist circumference in males (**A**) and waist-to-hip (WTH) ratio in females (**B**).

In males, the best correlation was confirmed for waist circumference (Figure 3A), while in females it was found for the waist-to-hip ratio (Figure 3B). In females, waist circumference showed a trend that did not reach significance, probably due to the limited number of subjects.

**Figure 4:**
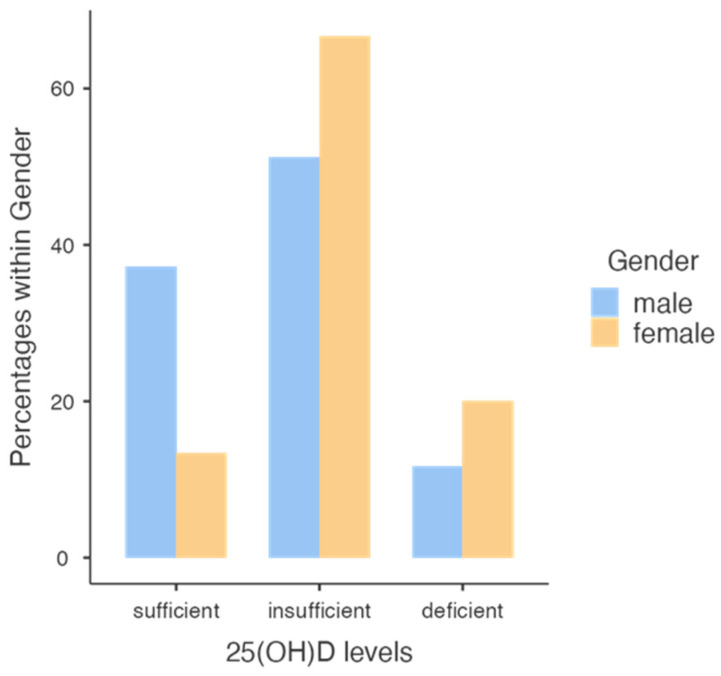
Level classes of 25 [OH] D concentration reached in males and females.

We evaluated the effects of administration of cholecalciferol in the two genders at follow up and we observed that only 38% of males and 17% of females reached normal values, particularly in subjects with higher VAT (Figure 4).

Note that a higher percentage of males reached a normal vitamin D level compared with females.

**Figure 5:**
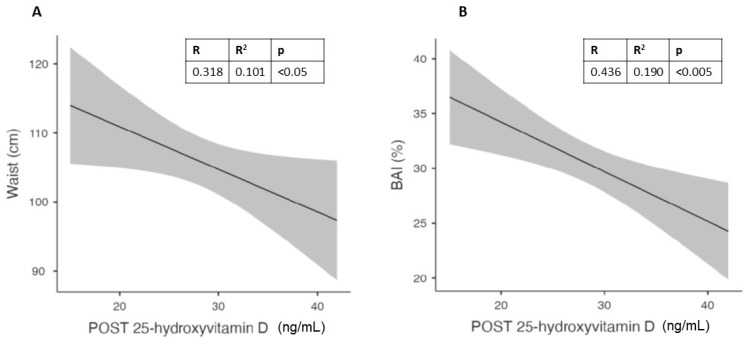
Correlation in males between vitamin D levels after 6 months of cholecalciferol and Waist circumference (**A**) and Body Adiposity Index (BAI) (**B**).

To evaluate the best predictors of vitamin D levels after 6 months of 50,000 IU/month of cholecalciferol in different genders, we observed in males a relationship between vitamin D levels at follow up and waist circumference at baseline (Figure 5A). In the same gender, we observed a better correlation between vitamin D levels and the body adiposity index (BAI; Figure 5B).

**Figure 6:**
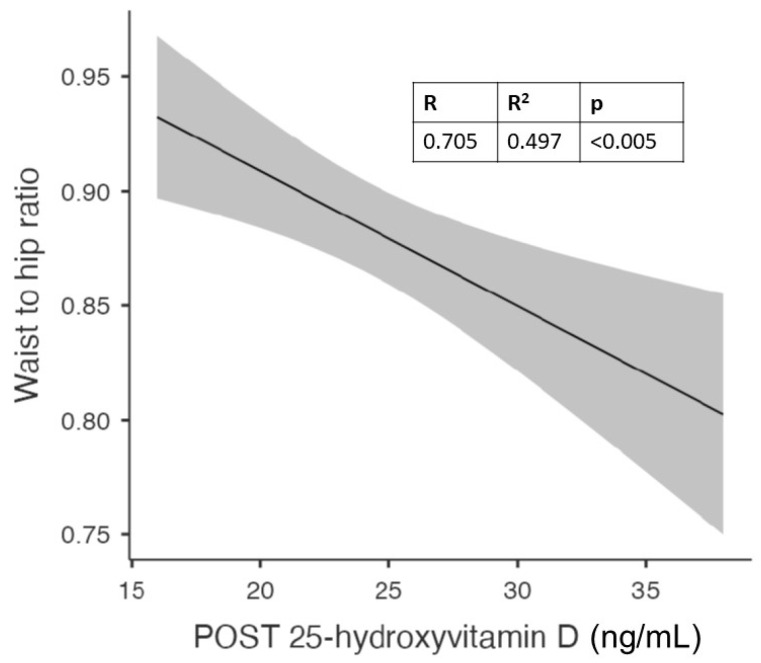
Correlation between waist-to-hip (WTH) ratio and vitamin D levels after 6 months of cholecalciferol in females.

In females, the best predictor of the vitamin D level at the end of the study was the waist-to-hip ratio (Figure 6).

On the basis of these correlations, we evaluated potential cut-off values that could predict the increase in vitamin D. Using the best predictors of vitamin D levels, we performed an ROC analysis in a subgroup of subjects with baseline vitamin D less than 20 ng/mL, reaching normal values above 30 ng/mL at the end of the study. We performed this analysis only in males, due to the limited number of females in this subgroup.

**Figure 7:**
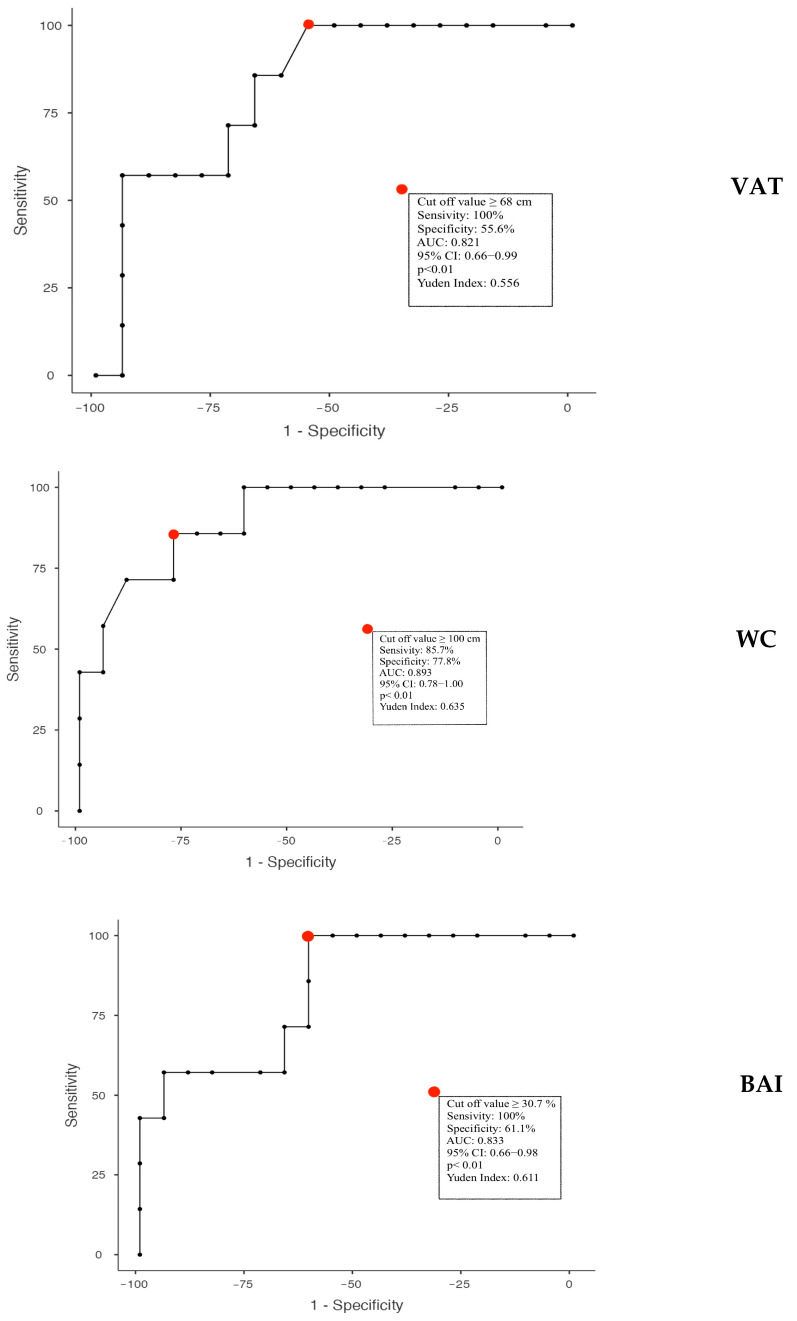

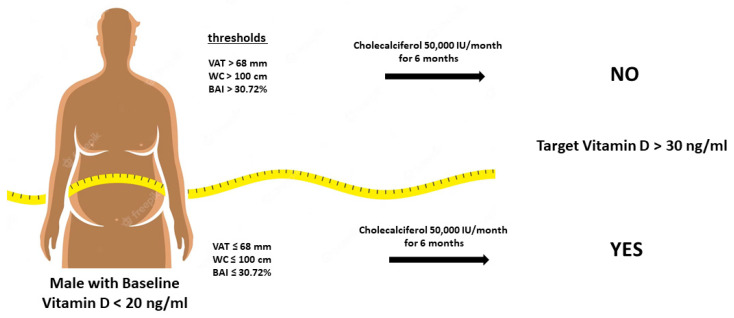
Calculated thresholds through the analysis of ROC curves of anthropometric and ultrasound parameters to predict the effect of vitamin D administration in obese subjects. VAT: Visceral Adipose Tissue; WC: Waist Circumference; BAI: Body Adiposity Index.

In males, we found the best negative value predictor for VAT ≤ 68 mm (Youden’s index: 0.556; AUC: 0.821; 95% CI: 0.66–0.99; *p* < 0.01), for waist circumference ≤ 100 cm (Youden’s index: 0.635; AUC: 0.893; 95% CI: 0.78–1.00; *p* < 0.01), and for BAI ≤ 30.72% (Youden’s index: 0.611; AUC: 0.833; 95% CI: 0.66–0.98; *p* < 0.01) (Figure 7).

## 5. Discussion

Vitamin D deficiency and insufficiency are widely prevalent across the globe [1]. The causes of vitamin D deficiency include insufficient outdoor physical activity leading to reduced sun exposure, inadequate dietary intake of vitamin D, obesity, and particularly, abdominal obesity [2,3]. Expression of the vitamin D receptor (VDR) gene and genes coding for enzymes that metabolize vitamin D has been observed in adipocytic cells. These observations suggest a high impact of vitamin D on the regulation of target genes in adipose tissue through endocrine, autocrine, and paracrine mechanisms [38]. Furthermore, VDR gene expression in visceral fat is dependent on 25(OH)D concentrations [39].

Therefore, vitamin D regulates the metabolic processes of adipose tissue, which, in turn, functions both as a deposit of vitamin D [40] and as a buffering system for the slow release of the molecule in order to reduce the uncontrolled synthesis of its active form, 1,25(OH)2D [17].

The relationship between vitamin D and adipose tissue is intricate, and the role of adipose tissue in regulating circulating vitamin D levels is not clear. Vitamin D is lipophilic and previous studies have demonstrated its accumulation in adipose tissue using a radioisotope approach [40,41]. Several studies suggest that the modulation of vitamin D and its effects in adipose tissue depends on the degree of obesity and the levels of adipose deposits [20] and that adiposity loss improves circulating 25(OH)D levels [42]. Moreover, the amount of vitamin D present in subcutaneous adipose tissue varies substantially, ranging from ~4 to ~500 ng/g, suggesting large individual variability and for an individual weighing 100 kg, with 40% body fat, this may equate to 160–20,000 mcg of vitamin D3 [43]. It has been shown that vitamin D and 25(OH)D stored in adipose tissue after 3 to 5 years of vitamin D supplementation may have a clinically relevant effect on the serum 25(OH)D level the following year [44].

On the other hand, previous research has demonstrated that individuals with obesity and abdominal obesity are at a higher risk of having inadequate or deficient serum vitamin D levels. Several authors have noted a significant inverse correlation between abdominal obesity and serum vitamin D levels [4,5,6,7]. Various theories have been proposed to explain the inverse relationship between vitamin D and adipose tissue, such as volumetric dilution. Some authors have observed that the increased adipose mass in obese individuals acts as a reservoir for vitamin D. This increased reservoir may require higher amounts of vitamin D to saturate it, potentially leading to inadequate serum vitamin D levels in obese individuals [22,23]. Alternatively, adipose tissue may sequester vitamin D, reducing its bioavailability [24]. Other authors suggest that reduced levels of 25 [OH] Dare associated with an increase in 1,25-dihydroxyvitamin D levels in obese individuals [25,26]. Unfortunately, none of these theories comprehensively explain the relationship between adipose tissue and circulating vitamin D levels.

In this study, we aimed to investigate the relationship between visceral adipose tissue and vitamin D levels, particularly exploring a potential threshold that determines the storage or sequestration of vitamin D using adipose tissue. In addition, we wanted to verify the correlation between ultrasound and anthropometric parameters to identify potential surrogates for the evaluation of visceral fat tissue, as previously performed by others [8,31].

We observed a significant correlation between follow-up vitamin D levels and waist circumference, hip circumference, and visceral adipose tissue in the overall population. These results suggest that abdominal obesity, specifically visceral adipose tissue, may play a crucial role in vitamin D homeostasis, as previously suggested by others [8,45].

Upon analyzing the data separately by gender, we found gender-specific differences in body fat distribution. Males exhibited a higher weight and waist-to-hip ratio, while females had a higher body adiposity index (BAI) and subcutaneous adipose tissue (SAT) thickness. Waist circumference in males and the waist-to-hip ratio in females showed the strongest correlations with vitamin D levels. Notably, the correlation between vitamin D levels and waist circumference in females did not reach significance, likely due to the limited number of subjects.

The further analysis of the effects of cholecalciferol supplementation revealed that a smaller proportion of males (38%) and females (17%) achieved normal vitamin D levels, particularly among subjects with higher visceral adipose tissue. We identified waist circumference at baseline as a predictor of vitamin D levels in males, while BAI showed a better correlation in this gender. In females, the waist-to-hip ratio was the best predictor of vitamin D levels at the end of the study. These findings suggest that different anthropometric variables may influence vitamin D status in males and females.

To establish potential cut-off values for predicting the increase in vitamin D levels, we performed a receiver operating characteristic (ROC) analysis in a subgroup of males with baseline vitamin D levels below 20 ng/mL, reaching normal values above 30 ng/mL at the end of the study. VAT, waist circumference, and BAI were identified as negative predictors in this subgroup.

Our findings suggest that in visceral adipose tissue, vitamin D is stored, but may become trapped, leading to its reduced availability for metabolic processes. These results support the hypothesis that adipose tissue acts both as a potential reservoir and sequestration site for vitamin D.

The strong correlation between visceral adipose tissue and vitamin D levels highlights the significance of adipose tissue in influencing vitamin D levels. The storage or sequestration of vitamin D in adipose tissue may have implications for individuals with obesity or abdominal obesity, as they are more likely to have insufficient or deficient vitamin D levels. This finding aligns with previous studies that have shown an inverse relationship between abdominal obesity and vitamin D levels [5,6,7].

The gender-specific differences observed in our study add further complexity to the relationship between vitamin D and adipose tissue. Males demonstrated stronger correlations between vitamin D levels and waist circumference, while females showed a stronger correlation with the waist-to-hip ratio. These differences may be attributed to variations in body fat distribution between genders. Further investigations are warranted to explore the underlying mechanisms and potential hormonal influences that contribute to these gender-specific associations.

Our study also examined the effects of cholecalciferol supplementation on vitamin D levels. Despite the supplementation, a considerable proportion of the subjects did not reach normal vitamin D levels, particularly those with higher visceral adipose tissue. This supports the evidence that the presence of excess adipose tissue may prevent the utilization or release of vitamin D, leading to suboptimal response to supplementation. Therefore, reducing visceral adipose tissue through lifestyle interventions [46] or weight loss strategies may be important for optimizing vitamin D status in individuals with obesity. For example, it has been demonstrated that physical activity is a powerful stimulus for lipid mobilization [42,47] and consensually releases vitamin D ‘trapped’ in adipocytes and greater serum 25(OH)D concentrations have been reported in individuals who self-report higher physical activity [48,49,50].

The identification of potential cut-off values for predicting the increase in vitamin D levels provides practical insights for clinical practice. In our subgroup analysis, specific thresholds of visceral adipose tissue, waist circumference, and BAI were associated with an improvement in vitamin D status. These cut-off values can be utilized as clinical indicators to identify individuals who may benefit from targeted interventions to enhance vitamin D levels and suggest the threshold that separates accumulation and sequestration. These results are consistent with previous experiments that have highlighted the need to significantly increase the vitamin D dose in specific situations in order to bring the levels back within the normal range [19].

It is worth noting that our study has certain limitations. Firstly, the sample size was relatively small, particularly in the subgroup analysis, which may have influenced the statistical power and generalizability of the results. Additionally, our study focused on a specific population with a high burden of comorbidities, which may limit the extrapolation of findings to other populations. Future studies with larger sample sizes and diverse populations are warranted to validate and extend our findings.

In conclusion, our study provides evidence for a significant correlation between visceral adipose tissue and vitamin D levels. The results suggest that visceral adipose tissue may sequester vitamin D, leading to reduced availability for metabolic processes. Abdominal obesity, especially visceral adipose tissue, appears to be associated with lower vitamin D levels, reinforcing the importance of maintaining a healthy body composition for optimal vitamin D status. Moreover, the relationship between obesity and vitamin D is intricate and bidirectional. Obesity can contribute to vitamin D deficiency, and vitamin D deficiency may exacerbate certain health risks associated with obesity. It is essential for healthcare professionals to consider these factors when assessing the health of obese individuals and to tailor interventions accordingly. Regular monitoring of vitamin D levels and appropriate supplementation, if necessary, can be crucial in managing the health of obese individuals.

Further research is needed to elucidate the underlying mechanisms and explore potential interventions to improve vitamin D status in individuals with obesity or abdominal obesity.

## Figures and Tables

**Table 1:** Baseline anthropometric parameters of the population.

Descriptive Analyses	Mean	Median	SD
Age (years)	72.6	74.5	11.22
Weight (Kg)	85.28	85	16.74
BMI (Kg/m^2^)	29.13	28.04	5.22
Waist circumference (cm)	104.37	102.5	12.81
Hip (cm)	111.46	110	12.48
BAI (%)	32.07	31.04	6.76
Waist-to-hip ratio	936	951	0.04
Waist-to-height ratio	611	606	0.07
SAT mean (mm)	18.2	15.3	8.15
VAT (mm)	70.06	66.9	26.86
PPAT (mm)	14.17	12	6.86
PRAT (mm)	9.52	8	7.04
25-hydroxyvitamin D (ng/mL)	17.59	18	5.59
Calcium (mg/dL)	9.33	9.35	0.44

BMI: body mass index; BAI: body adiposity index; SAT: subcutaneous adipose tissue; VAT: visceral adipose tissue; PPAT: preperitoneal adipose tissue; PRAT: prerenal adipose tissue.

**Table 2:** Baseline anthropometric parameters of the population grouped according to gender.

	Group	N	Mean	SD	*p*
Age (years)	males	43	72.83	11.56	0.791
	females	15	71.93	10.55	
Weight (Kg)	males	43	88.34	15.98	0.017
	females	15	76.53	16.27	
Height (m)	males	43	1.74	0.06	<0.001
	females	15	1.63	0.07	
BMI (Kg/m^2^)	males	43	29.26	5.44	0.74
	females	15	28.74	4.7	
Waist circumference (cm)	males	43	105.77	11.88	0.158
	females	15	100.33	14.87	
Hip circumference (cm)	males	43	110.83	12.3	0.521
	females	15	113.26	13.26	
BAI (%)	males	43	30.47	6.36	0.002
	females	15	36.65	5.83	
Waist-to-hip ratio	males	43	955	0.03	< 0.001
	females	15	883	0.04	
Vitamin D (ng/mL)	males	43	17.9	5.9	0.46
	females	15	16.7	4.8	
SAT mean (mm)	males	43	16.1	7.53	<0.001
	females	15	24.1	7.01	
VAT (mm)	males	43	70.63	28.53	0.787
	females	15	68.43	22.16	
PPAT (mm)	males	43	14.17	7.12	0.999
	females	15	14.17	6.3	
PRAT (mm)	males	42	9.03	7.14	0.384
	females	15	10.89	6.81	

BMI: body mass index; BAI: body adiposity index; SAT: subcutaneous adipose tissue; VAT: visceral adipose tissue; PPAT: preperitoneal adipose tissue; PRAT: prerenal adipose tissue.

## Data Availability

The datasets used and/or analyzed during the current study are available from the corresponding author upon a reasonable request.

## References

[1] Bouillon R., Norman A.W., Lips P. (2007). Vitamin D deficiency. N. Engl. J. Med..

[2] Earthman C., Beckman L., Masodkar K., Sibley S. (2012). The link between obesity and low circulating 25-hydroxyvitamin D concentrations: Considerations and implications. Int. J. Obes..

[3] Brock K., Huang W.-Y., Fraser D., Ke L., Tseng M., Stolzenberg-Solomon R., Peters U., Ahn J., Purdue M., Mason R. (2010). Low vitamin D status is associated with physical inactivity, obesity and low vitamin D intake in a large US sample of healthy middle-aged men and women. J. Steroid Biochem. Mol. Biol..

[4] da Silva E.M.S., Pinho H.S., Rodrigues I.G., Pinho C.P.S. (2019). Asociación entre los niveles séricos de vitamina D y las alteraciones cardiometabólicas. Nutr. Clínica Dietética Hosp..

[5] Zaki M., Kamal S., Basha W.A., Youness E., Ezzat W., El-Bassyouni H., Amr K. (2017). Association of vitamin D receptor gene polymorphism (VDR) with vitamin D deficiency, metabolic and inflammatory markers in Egyptian obese women. Genes Dis..

[6] Farrell S.W., Willis B.L. (2012). Cardiorespiratory fitness, adiposity, and serum 25-dihydroxyvitamin D levels in women: The Cooper Center Longitudinal Study. J. Women’s Health.

[7] da Silva S., Maria E., Pinho, Sabino H., Rodrigues, GalvÃ£o1 I., Pinho, Sabino C.P. (2019). Association between serum vitamin D levels and cardiometabolic alterations. Nutr. Clín. Diet. Hosp..

[8] Hajhashemy Z., Foshati S., Saneei P. (2022). Relationship between abdominal obesity (based on waist circumference) and serum vitamin D levels: A systematic review and meta-analysis of epidemiologic studies. Nutr. Rev..

[9] Ritchie S., Connell J. (2007). The link between abdominal obesity, metabolic syndrome and cardiovascular disease. Nutr. Metab. Cardiovasc. Dis..

[10] Aggoun Y. (2007). Obesity, metabolic syndrome, and cardiovascular disease. Pediatr. Res..

[11] Zhong P., Tan S., Zhu Z., Zhu Z., Liang Y., Huang W., Wang W. (2023). Normal-weight central obesity and risk of cardiovascular and microvascular events in adults with prediabetes or diabetes: Chinese and British cohorts. Diabetes/Metab. Res. Rev..

[12] Hulsmans M., Van Dooren E., Mathieu C., Holvoet P. (2012). Decrease of miR-146b-5p in monocytes during obesity is associated with loss of the anti-inflammatory but not insulin signaling action of adiponectin. PLoS ONE.

[13] Kouli G.-M., Panagiotakos D.B., Kyrou I., Georgousopoulou E.N., Chrysohoou C., Tsigos C., Tousoulis D., Pitsavos C. (2017). Visceral adiposity index and 10-year cardiovascular disease incidence: The ATTICA study. Nutr. Metab. Cardiovasc. Dis..

[14] Dalle Carbonare L., Valenti M.T., Del Forno F., Piacentini G., Pietrobelli A. (2018). Vitamin D daily versus monthly administration: Bone turnover and adipose tissue influences. Nutrients.

[15] Pecoraro L., Nisi F., Serafin A., Antoniazzi F., Dalle Carbonare L., Piacentini G., Pietrobelli A. (2022). Vitamin d supplementation in the assessment of cardiovascular risk factors in overweight and obese children. Med. Sci..

[16] Zakharova I., Klimov L., Kuryaninova V., Nikitina I., Malyavskaya S., Dolbnya S., Kasyanova A., Atanesyan R., Stoyan M., Todieva A. (2019). Vitamin D insufficiency in overweight and obese children and adolescents. Front. Endocrinol..

[17] Płudowski P., Kos-Kudła B., Walczak M., Fal A., Zozulińska-Ziółkiewicz D., Sieroszewski P., Peregud-Pogorzelski J., Lauterbach R., Targowski T., Lewiński A. (2023). Guidelines for preventing and treating vitamin D deficiency: A 2023 update in Poland. Nutrients.

[18] Bilezikian J.P., Formenti A.M., Adler R.A., Binkley N., Bouillon R., Lazaretti-Castro M., Marcocci C., Napoli N., Rizzoli R., Giustina A. (2021). Vitamin D: Dosing, levels, form, and route of administration: Does one approach fit all?. Rev. Endocr. Metab. Disord..

[19] Pludowski P. (2023). Supplementing Vitamin D in Different Patient Groups to Reduce Deficiency. Nutrients.

[20] Szymczak-Pajor I., Miazek K., Selmi A., Balcerczyk A., Śliwińska A. (2022). The action of vitamin D in adipose tissue: Is there the link between vitamin D deficiency and adipose tissue-related metabolic disorders?. Int. J. Mol. Sci..

[21] Ding C., Gao D., Wilding J., Trayhurn P., Bing C. (2012). Vitamin D signalling in adipose tissue. Br. J. Nutr..

[22] Carrelli A., Bucovsky M., Horst R., Cremers S., Zhang C., Bessler M., Schrope B., Evanko J., Blanco J., Silverberg S.J. (2017). Vitamin D storage in adipose tissue of obese and normal weight women. J. Bone Miner. Res..

[23] Drincic A.T., Armas L.A., Van Diest E.E., Heaney R.P. (2012). Volumetric dilution, rather than sequestration best explains the low vitamin D status of obesity. Obesity.

[24] Wortsman J., Matsuoka L.Y., Chen T.C., Lu Z., Holick M.F. (2000). Decreased bioavailability of vitamin D in obesity. Am. J. Clin. Nutr..

[25] Hey H., Stokholm K., Lund B., Sørensen O. (1982). Vitamin D deficiency in obese patients and changes in circulating vitamin D metabolites following jejunoileal bypass. Int. J. Obes..

[26] Bell N.H., Greene A., Epstein S., Oexmann M.J., Shaw S., Shary J. (1985). Evidence for alteration of the vitamin D-endocrine system in blacks. J. Clin. Investig..

[27] Konradsen S., Ag H., Lindberg F., Hexeberg S., Jorde R. (2008). Serum 1, 25-dihydroxy vitamin D is inversely associated with body mass index. Eur. J. Nutr..

[28] Moan J., Lagunova Z., Lindberg F.A., Porojnicu A.C. (2009). Seasonal variation of 1, 25-dihydroxyvitamin D and its association with body mass index and age. J. Steroid Biochem. Mol. Biol..

[29] Lagunova Z., Porojnicu A.C., Vieth R., Lindberg F.A., Hexeberg S., Moan J. (2011). Serum 25-hydroxyvitamin D is a predictor of serum 1, 25-dihydroxyvitamin D in overweight and obese patients. J. Nutr..

[30] Vlachos I.S., Hatziioannou A., Perelas A., Perrea D.N. (2007). Sonographic assessment of regional adiposity. Am. J. Roentgenol..

[31] Pimanov S., Bondarenko V., Makarenko E. (2020). Visceral fat in different locations assessed by ultrasound: Correlation with computed tomography and cut-off values in patients with metabolic syndrome. Clin. Obes..

[32] Kotsis V., Tsioufis K., Antza C., Seravalle G., Coca A., Sierra C., Lurbe E., Stabouli S., Jelakovic B., Redon J. (2018). Obesity and cardiovascular risk: A call for action from the European Society of Hypertension Working Group of Obesity, Diabetes and the High-risk Patient and European Association for the Study of Obesity: Part B: Obesity-induced cardiovascular disease, early prevention strategies and future research directions. J. Hypertens..

[33] Oda E. (2006). New criteria forobesity disease’in Japan. Circ. J..

[34] Bergman R.N., Stefanovski D., Buchanan T.A., Sumner A.E., Reynolds J.C., Sebring N.G., Xiang A.H., Watanabe R.M. (2011). A better index of body adiposity. Obesity.

[35] Fang H., Berg E., Cheng X., Shen W. (2018). How to best assess abdominal obesity. Curr. Opin. Clin. Nutr. Metab. Care.

[36] Boone S.C., Van Smeden M., Rosendaal F.R., Le Cessie S., Groenwold R.H., Jukema J.W., Van Dijk K.W., Lamb H.J., Greenland P., Neeland I.J. (2022). Evaluation of the value of waist circumference and metabolomics in the estimation of visceral adipose tissue. Am. J. Epidemiol..

[37] Smith U. (2015). Abdominal obesity: A marker of ectopic fat accumulation. J. Clin. Investig..

[38] Park C.Y., Han S.N. (2021). The role of vitamin D in adipose tissue biology: Adipocyte differentiation, energy metabolism, and inflammation. J. Lipid Atheroscler..

[39] Yuzbashian E., Asghari G., Hedayati M., Zarkesh M., Mirmiran P., Khalaj A. (2019). Determinants of vitamin D receptor gene expression in visceral and subcutaneous adipose tissue in non-obese, obese, and morbidly obese subjects. J. Steroid Biochem. Mol. Biol..

[40] Rosenstreich S.J., Rich C., Volwiler W. (1971). Deposition in and release of vitamin D 3 from body fat: Evidence for a storage site in the rat. J. Clin. Investig..

[41] Mawer E.B., Backhouse J., Holman C.A., Lumb G., Stanbury S. (1972). The distribution and storage of vitamin D and its metabolites in human tissues. Clin. Sci..

[42] Gangloff A., Bergeron J., Lemieux I., Després J.-P. (2016). Changes in circulating vitamin D levels with loss of adipose tissue. Curr. Opin. Clin. Nutr. Metab. Care.

[43] Didriksen A., Burild A., Jakobsen J., Fuskevåg O.M., Jorde R. (2015). Vitamin D3 increases in abdominal subcutaneous fat tissue after supplementation with vitamin D3. Eur. J. Endocrinol..

[44] Martinaityte I., Kamycheva E., Didriksen A., Jakobsen J., Jorde R. (2017). Vitamin D stored in fat tissue during a 5-year intervention affects serum 25-hydroxyvitamin D levels the following year. J. Clin. Endocrinol. Metab..

[45] Pourshahidi L.K. (2015). Vitamin D and obesity: Current perspectives and future directions. Proc. Nutr. Soc..

[46] Hengist A., Perkin O., Gonzalez J., Betts J., Hewison M., Manolopoulos K., Jones K., Koulman A., Thompson D. (2019). Mobilising vitamin D from adipose tissue: The potential impact of exercise. Nutr. Bull..

[47] Thompson D., Karpe F., Lafontan M., Frayn K. (2012). Physical activity and exercise in the regulation of human adipose tissue physiology. Physiol. Rev..

[48] Gangloff A., Bergeron J., Pelletier-Beaumont E., Nazare J., Smith J., Borel A., Lemieux I., Tremblay A., Poirier P., Alméras N. (2015). Effect of adipose tissue volume loss on circulating 25-hydroxyvitamin D levels: Results from a 1-year lifestyle intervention in viscerally obese men. Int. J. Obes..

[49] Reinehr T., de Sousa G., Alexy U., Kersting M., Andler W. (2007). Vitamin D status and parathyroid hormone in obese children before and after weight loss. Eur. J. Endocrinol..

[50] Chin K., Zhao D., Tibuakuu M., Martin S.S., Ndumele C.E., Florido R., Windham B.G., Guallar E., Lutsey P.L., Michos E.D. (2017). Physical activity, vitamin D, and incident atherosclerotic cardiovascular disease in whites and blacks: The ARIC study. J. Clin. Endocrinol. Metab..

